# Multisensory Integration and Internal Models for Sensing Gravity Effects in Primates

**DOI:** 10.1155/2014/615854

**Published:** 2014-07-01

**Authors:** Francesco Lacquaniti, Gianfranco Bosco, Silvio Gravano, Iole Indovina, Barbara La Scaleia, Vincenzo Maffei, Myrka Zago

**Affiliations:** ^1^Centre of Space Bio-Medicine, University of Rome Tor Vergata, Via Montpellier 1, 00133 Rome, Italy; ^2^Department of Systems Medicine, University of Rome Tor Vergata, Via Montpellier 1, 00133 Rome, Italy; ^3^Laboratory of Neuromotor Physiology, IRCCS Santa Lucia Foundation, Via Ardeatina 306, 00179 Rome, Italy

## Abstract

Gravity is crucial for spatial perception, postural equilibrium, and movement generation. The vestibular apparatus is the main sensory system involved in monitoring gravity. Hair cells in the vestibular maculae respond to gravitoinertial forces, but they cannot distinguish between linear accelerations and changes of head orientation relative to gravity. The brain deals with this sensory ambiguity (which can cause some lethal airplane accidents) by combining several cues with the otolith signals: angular velocity signals provided by the semicircular canals, proprioceptive signals from muscles and tendons, visceral signals related to gravity, and visual signals. In particular, vision provides both static and dynamic signals about body orientation relative to the vertical, but it poorly discriminates arbitrary accelerations of moving objects. However, we are able to visually detect the specific acceleration of gravity since early infancy. This ability depends on the fact that gravity effects are stored in brain regions which integrate visual, vestibular, and neck proprioceptive signals and combine this information with an internal model of gravity effects.

## 1. Introduction

Intuitively, sensing gravity effects should be a trivial problem for a complex nervous system such as our own. On the one hand, direction and magnitude of gravity are quasi-constant on Earth. Thus, gravitational acceleration varies by <1% by changing latitude or altitude, while the vertical deflection is <0.05°. On the other hand, our nervous system is computationally high-powered, being endowed with ≈10^11^ neurons interconnected via ≈10^15^ synapses. All axons pieced together would cover the distance between the Earth and the Moon (about 400.000 km). One would assume that we are able to monitor gravity directly by means of our sensory systems, but this is not the case. As we shall review in this paper, gravity effects are only extrapolated indirectly by the brain by combining multisensory information with internal models, that is, with neural processes which mimic a physical event.

Sensing and coping with gravity is crucial for space perception, control of upright posture, and generation of movements. Indeed, gravity provides a unique reference axis to which we can anchor body orientation and monitor orientation changes. Gravity effects on limb and body movements are two-sided, insofar as gravity acts both as a perturbing force that must be counteracted to avoid falling down and as a facilitating force which allows walking and running via the ground contact forces.

## 2. Vestibular Information

The vestibular receptors lie inside the labyrinth of the temporal bone. Somewhat similar sensors evolved first in invertebrates and then in vertebrates about 500 Myrs ago [1]. The vestibular apparatus acts as an inertial navigation system, including in each ear three semicircular canals oriented roughly orthogonal to each other and two otolithic organs, the sacculus and utriculus with sensory epithelia oriented roughly vertically and horizontally, respectively [2]. The vestibular sensors function as accelerometers, the semicircular canals transducing angular accelerations (roll, yaw, and pitch) and the otoliths transducing linear accelerations. Head acceleration bends the cilia of the hair cells in the sensory organs, resulting in a change of the membrane potential and synaptic transmission of the neurons of the vestibular ganglion innervating the receptors. The signals from the vestibular neurons carry information about head velocity and acceleration to the vestibular nuclei in the brain stem. In turn, signals from these nuclei are relayed and processed in several regions of the brain and spinal cord, giving rise to sensations and movements [3].

**Figure 1:**
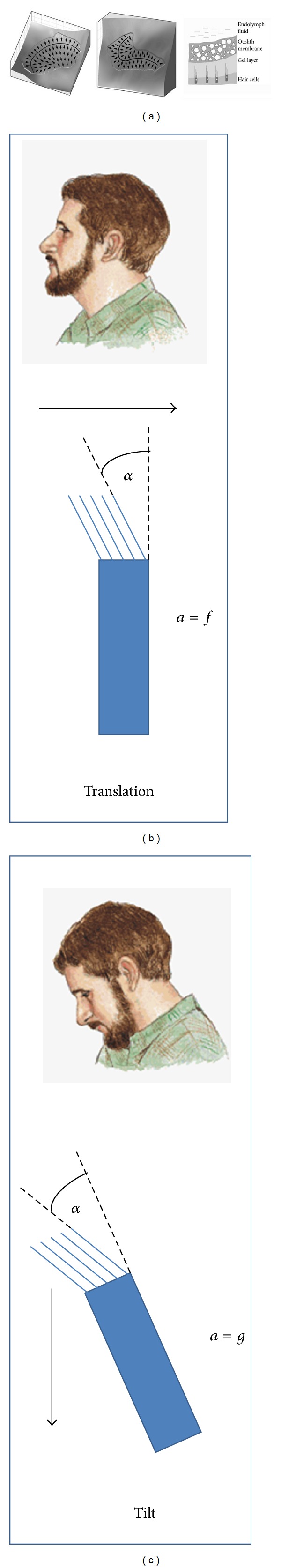
(a) Otolith organs. Left: utricle. Center: saccule. Arrows indicate the local on-directions of the hair cells; thick black lines indicate the striola. Right: cross-section through the otolith membrane showing the different layers. Licensed under the Creative Commons Attribution-Share (authors: Thomas Haslwanter and Rudi Jaeger). (b)-(c) Tilt-translation ambiguity of otolith receptors. (b) The upright head is accelerated backward. (c) The head is tilted forward. These two gravitoinertial accelerations cannot be discriminated by the otolith sensory neurons.

The widely distributed polarities of response of the otolith receptors in the maculae allow monitoring acceleration vectors in any arbitrary direction (Figure 1(a)). These receptors are extremely sensitive, being able to detect displacements of the cilia as small as 0.3 nm (typical atomic diameter) and correspondingly small accelerations. In fact, the receptors in the maculae respond to the projection of an applied force (or acceleration). Thus, the component of gravitational acceleration projected on the saccular macula is *g* cos⁡ *α*, where *α* is the angle of tilt of the head relative to the gravity direction, whereas the component of gravitational acceleration projected on the utricle macula is *g* sin *α*. Gravity accelerates the body downwards and is opposed by the ground contact forces. These contact forces are transmitted to all body segments and to the head, where they are monitored by the otolith receptors. These receptors respond to a tilt of the head relative to gravity, but in general they cannot provide a unique measurement of gravity effects. As any accelerometer, also the sacculus and utricle respond to net gravitoinertial accelerations, and they cannot distinguish between the gravitational and the inertial component. For instance, otolith afferents cannot distinguish whether we are accelerating backward (Figure 1(b)) or tilting the head forward (Figure 1(c)). This is because the effect of gravity is locally indistinguishable from the effect of a linear acceleration of the reference system [8]. In fact, the otolith afferents signal the net gravitoinertial acceleration (*a*) resulting from the vector difference between the gravity vector (*g*) and the linear acceleration vector (*f*):

(1) $$\begin{array}{c} a=g-f. \end{array}$$

All vectors are time-varying, referred to head-fixed coordinates of the vestibular sensors.

This intrinsic ambiguity can give rise to perceptual illusions which become extremely dangerous under some specific conditions. For instance, during takeoff, an airplane pilot may sense an erroneously high value of upward pitch, because the resultant of the vector sum of gravity and backward inertial acceleration is misperceived as the actual orientation relative to the vertical. Under conditions of low visibility and without the aid of instruments, the pilot may then attempt to correct the aircraft attitude by pitching downward, with the risk of impacting the ground. Spatial disorientations originating from sensory ambiguities of this kind are often involved in severe aviation accidents [9].

Under usual conditions, however, we have no difficulty in sensing the orientation of the head relative to the vertical, even with the eyes closed and in the presence of appreciable accelerations, provided the latter have short duration (as those of a car or train). This is because the ambiguity can be solved by the brain using a variety of “tricks.” First, the brain filters the otolith signals, so that the low-frequency (longer lasting) signals are automatically interpreted as a change in the tilt angle of the head relative to gravity [10]. Conversely, high-frequency (shorter lasting) signals are interpreted as related to a linear acceleration. Also the perceptual illusion of the aircraft pilot mentioned above is consistent with the frequency segregation hypothesis. A prolonged linear acceleration (such as that at airplane takeoff) is a very rare event; when it occurs, it is interpreted erroneously as a tilt relative to gravity. Notice, however, that, unlike the output of a simple low-pass filter, the phase of perceived tilt has been shown to be relatively constant across a broad frequency range [3, 11].

A second “trick” used to disambiguate gravitoinertial acceleration consists in combining the otolith signals with those of the semicircular canals [11–13], just as the man-made inertial systems which combine accelerometers and gyroscopes. When we turn our heads, the semicircular canals integrate the angular acceleration and signal the corresponding angular velocity for frequencies above about 0.05 Hz [14]. Information about angular head velocity can then be used to keep track of changes in orientation of the gravity vector relative to the head [15, 16]. Formally,

(2) $$\begin{array}{c} \dot{g}=g\times \omega , \end{array}$$

where $\dot{g}$ denotes the time derivative of the gravity vector, *ω* denotes the angular velocity, and × denotes vector cross-product. An internal three-dimensional estimate of the gravity vector in head coordinates can then be obtained by integrating (2), if the initial conditions for *g* are known:

(3) $$\begin{array}{c} g=\int g\times \omega \;dt. \end{array}$$

Notice that the angular velocity that needs to be integrated in (3) is represented by the component parallel to the Earth horizon, because this component changes the orientation of the head relative to gravity. Given the estimate of *g* provided by (3), gravitoinertial accelerations can be disambiguated by solving (1).

**Figure 2:**
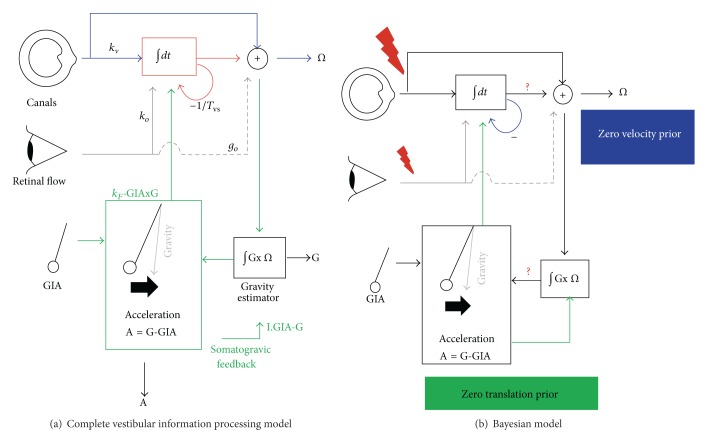
Model of visuovestibular processing proposed by Laurens and Angelaki [4]. (a) Blue lines, vestibular pathways; grey lines, visual pathways; green lines, inertial pathways. (b) Schematic model of Bayesian inference for vestibular processing. Black lines, deterministic model; lightning bolts, sources of noise; question marks, points of error accumulation; blue lines, influence of the zero velocity prior; green lines, influence of the zero translation prior; grey lines, incorporation of visual information (reproduced with permission from [4]).

A potential problem with the model outlined above is that the semicircular canals do not provide a reliable estimate of angular velocity at steady-state [14]. Errors in the estimate of *ω* would determine an error in the estimate of tilt relative to gravity provided by (3). A solution consists in correcting the errors by means of the so-called somatogravic feedback (Figure 2), which tends to align the estimate of the gravitational acceleration with the gravitoinertial acceleration [4, 5, 17]. In other words, the time-average of the gravitoinertial acceleration over several seconds yields an estimate of gravity orientation at low frequencies. The somatogravic effect can be incorporated in the model of (2) by including a low-pass filtered term to the tilt estimate, thereby canceling any drift [4]. The resulting equation is

(4) $$\begin{array}{c} \dot{g}=g\times \omega -\frac{g-a}{\tau }. \end{array}$$

The time constant *τ* controls the gain and phase of the *g* estimate when the otolith organs alone are activated, for example, by pure translation. Alternatively, the somatogravic feedback effects can be substituted by a Bayesian prior at zero translational acceleration [4, 17]. This prior is also compatible with the aviation illusion mentioned above. Indeed, while the correction due to the feedback or the prior is beneficial under normal conditions, it can result in the so-called somatogravic illusion [4, 9]. During translation, the tilt estimate increases over time as the estimated gravity moves towards the gravitoinertial acceleration. This causes a decrease in the translation estimate and in an aftereffect at the end of the translational acceleration.

**Figure 3:**
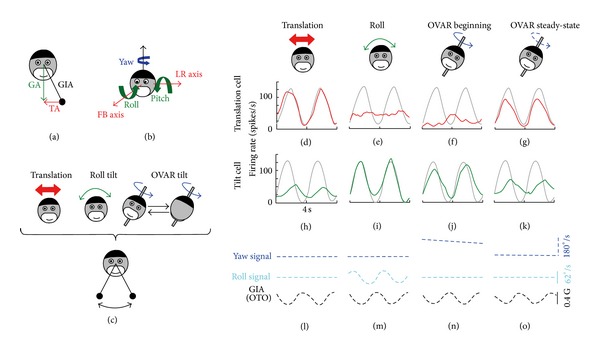
(a) Equivalence principle: the otolith organs are sensitive to the gravitoinertial acceleration (GIA), equal to the difference between the gravity vector (GA) and the translational acceleration (TA). (b) Naming conventions of the head's translation and rotation axes. FB, forward-backward; LR, leftward-rightward. (c) Representation of the motion protocols used by Laurens et al. [9]. GIA along the LR axis, represented by a swinging pendulum (bottom), is identical in the 3 protocols (translation, tilt, and off-vertical axis rotation [OVAR]). ((d)–(o)) Responses from a translation-selective cell (red) and a tilt-selective cell (green) during left-right (LR) translation ((d) and (h)), roll tilt ((e) and (i)), and constant velocity OVAR ((f), (g), (j), and (k)). (l), (m), (n), and (o) show the corresponding yaw velocity (detected by horizontal canals, blue), roll velocity (detected by vertical canals, cyan), and GIA along the LR axis (detected by otolith organs [OTO], black). Gray curves: fit to the LR translation response (shown in (d), translation cell) or the roll tilt response (shown in (i), tilt cell) (reproduced with permission from [5]).

Neural correlates of the operations described above have been discovered in the monkey by Angelaki and colleagues, who found that the neural computation of translation (*f*) occurs in the so-called Vestibular-only neurons of the vestibular nuclei, in the rostral portion of cerebellar fastigium and nodulus [18]. Neurons in these regions combine temporally processed signals from the canals and otoliths as predicted by the internal model hypothesis. Recently, neurons extracting gravity have been discovered in the cerebellum [5]. Laurens et al. identified a group of Purkinje cells in the caudal cerebellar vermis with responses that reflect an estimate of head tilt (Figure 3). These tilt-selective cells are complementary to the translation-selective Purkinje cells mentioned above, such that their population activities sum to the net gravitoinertial acceleration encoded by the otolith organs.

## 3. Multisensory Integration

As we remarked in the previous section, vestibular sensations result from composite signals, because the otolith signals are centrally combined with those of the semicircular canals already at the level of second-order sensory neurons in the vestibular nuclei of the brainstem. As far as gravity transduction is concerned, the vestibular signals are centrally combined with other sensory information, such as proprioceptive signals from muscle and tendon receptors, visceral signals (from the kidneys, vena cava, etc.), and visual signals. Vision, in particular, provides both static and dynamic (e.g., optic flow) signals about the orientation of the body relative to the vertical. Finally, also the so-called efference copy of motor signals (i.e., a copy of the motor commands sent by higher brain centers) and internal estimates of the body axis orientation [19] contribute to an estimate of body orientation. All these signals are centrally combined yielding accurate multisensory estimates about gravity direction. Indeed, in darkness an erect person makes errors <2° when aligning an initially tilted luminous bar with the expected direction of gravity [20].

Under normal light conditions of daily life, there are several visual cues which point to the direction of gravity [21]. Thus, trees are rooted downwards and grow vertically upwards, and the walls of the houses are also vertical, as are the chandeliers hanging from the ceiling. The visual reference to gravity is so strong that there exist tourist attraction places (so-called mystery spots) where some anomaly of the environment is exploited to provide the illusion that the gravity law is violated. For instance, in some places there is a strong slope of the terrain and trees grow slanted. Similar effects can be obtained with tilted walls in houses built for the purpose of creating such illusions. The slope angle distorts the perspective of the observer and may even create the perceptual illusion that a ball can roll upwards by itself. In Italy, such illusions can be felt inside the leaning house designed by Vicino Orsini at Bomarzo (see http://en.wikipedia.org/wiki/File:Bomarzo_parco_mostri_casa_pendente.jpg).

Except when some cue is so strong as to drive space perception by itself (a winner-take all situation), neural estimates of gravity direction normally are computed by the central nervous system as a weighted average of multicue information, including vestibular, visual, neck, and truncal signals, plus a prior distribution about head and body orientation based on experience [20, 22–24]. In Bayesian terms, the posterior estimate is obtained by combining noisy sensory measurements with a prior, each term being weighed inversely to its variance (noise [23]).

## 4. Visual Perception of Gravitational Acceleration

So far, we considered the problem of monitoring the direction of gravity. A different problem concerns monitoring its magnitude. How do we estimate the gravitational acceleration of an object in a visual scene? This situation occurs quite frequently, as when we experience the vision of objects in free-fall, projectile, or pendulum motion. In addition to object motion, also self-motion may involve visual stimuli (optic flow) accelerated by gravity, as when we fall or jump from a height. When confronted with gravity effects, the visual system faces a unique challenge. In contrast with body graviceptors (such as those of the vestibular system, muscle and tendon proprioceptors, and visceral organs), the visual system does not deal with physical gravity directly, but only with the acceleration of the retinal image. Whereas gravitational acceleration is constant at a given location, the corresponding retinal acceleration varies inversely with the viewing distance (distance between the observer and the scene). Therefore, the visual estimate of gravity effects on a target motion requires accurate estimates of both image acceleration and viewing distance. Both types of estimates are potentially problematic. Indeed, while the visual system is very accurate in velocity estimates, it is rather poor in acceleration estimates. In fact, the visual discrimination of acceleration is about 5 times worse than that of velocity [25]. Also, viewing distance may be difficult to assess. Eye vergence, accommodation, and stereo-disparity contribute to estimating viewing distance of target motion in three-dimensional space, but these cues are ineffective when the target is far (because of trigonometry) or when it moves on a two-dimensional video display (as in a videogame). Pictorial information—such as that provided by the presence of objects of known size (people, trees, houses, etc.) in the visual scene—also aids recovering an environmental reference and scale and thus allows the calibration of the retinal image [26].

**Figure 4:**
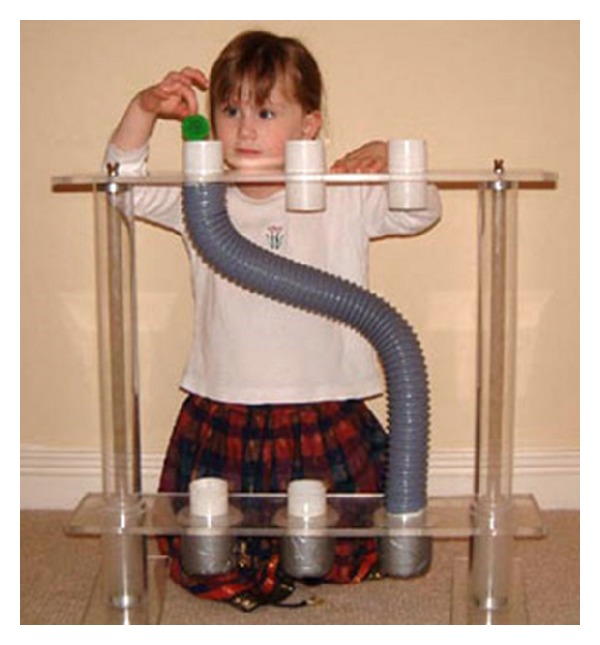
Test with curved tubes in preschoolers (reproduced with permission of Prof. Bruce Hood, University of Bristol).

An internal model of gravity effects represents a critical component of the visual estimates in addition to raw sensory signals. Indeed, gravity represents a special case of visual acceleration to which we are exposed since birth. Therefore, it is very likely that it has been internalized in the brain. In fact, it has been shown that gravity effects on a visual object are detected early in life [27]. Between 5 and 7 months of age, infants expect that an object moving down an inclined plane accelerates and an upwardly moving object decelerates and are surprised to see the effects of an artificial reversed gravity (i.e., objects decelerating while moving downwards and accelerating while moving upwards). Implicit expectation of gravity effects can generate striking judgment errors in preschoolers. Children around 2 years of age believe that a descending object always falls vertically downwards. Thus, when they are asked to find a ball that is dropped along a curved tube, they search directly under the point of fall rather than at the exit of the tube [28]. However, if the ball motion is artificially reversed so that the ball seems to rise upwards, all children solve the task perfectly (Figure 4). Notice that false beliefs about free-fall can still persist in adulthood (so-called naïve physics). For instance, several people without formal scientific background believe that heavier objects fall faster than lighter objects of the same size [6].

**Figure 5:**
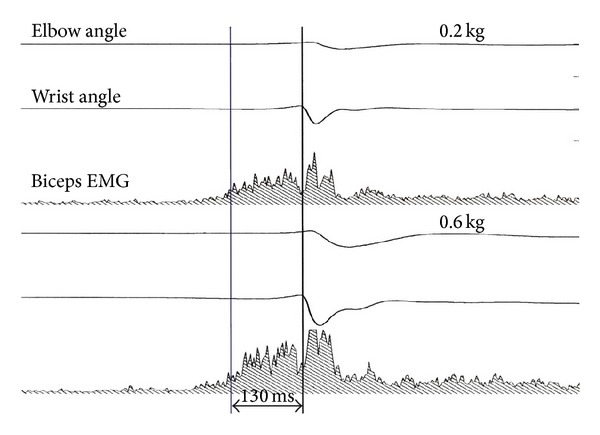
Catching balls of different weight. In different trials, a subject caught a 0.2 kg ball (upper panel) and a 0.6 kg ball (lower panel), dropped from a 1.2 m height. In each panel, traces from top to bottom correspond to elbow flexion angle, wrist flexion angle, and rectified electrical activity (EMG) of biceps muscle. The right-most vertical line denotes the time of impact of the ball on the hand. The left-most vertical line denotes the time of onset of the anticipatory EMG activity (reproduced with permission from [6]).

**Figure 6:**
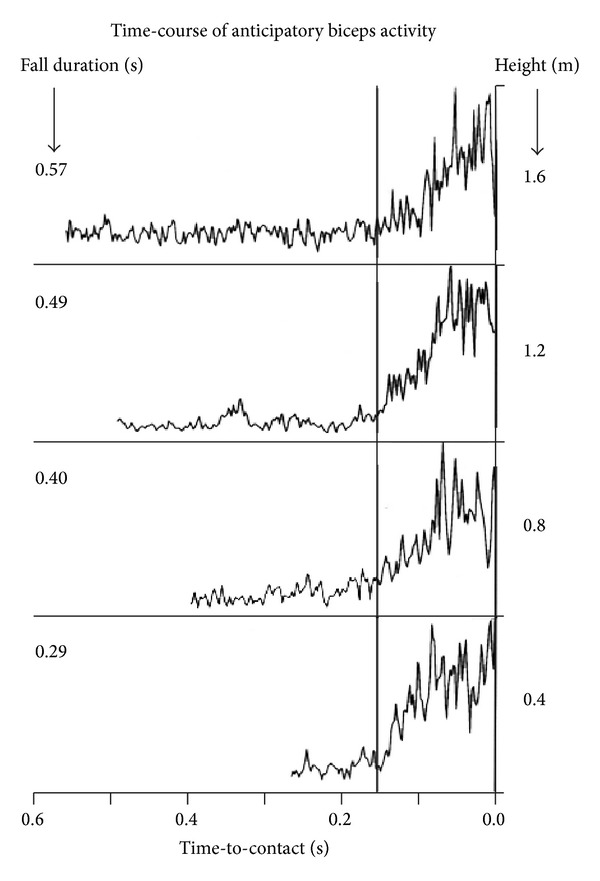
Time course of the EMG anticipatory responses of biceps. Traces correspond to the results obtained for catches of balls dropped from the heights indicated on the right (fall durations are indicated on the left). EMG traces have been scaled in amplitude to their maximum and aligned relative to collision time. Time axis indicates the time remaining prior to collision (reproduced with permission from [6]).

Strikingly, however, the motor system has an implicit knowledge of physics which is much better than that available to the cognitive system [6]. Daily life offers several examples of behavior demonstrating the implicit knowledge of physics and the anticipation of the effects of gravitational and centrifugal forces, for instance, when we try to keep equilibrium while riding a bike. Also the automatic motor responses evoked by seeing a falling object are programmed by the brain by taking into account the law of free-fall first formulated by Galileo Galilei. In a laboratory experiment (Figure 5), subjects were asked to catch with the hand a ball that was dropped vertically from 1.2 m height relative to the hand [6]. The ball could weigh 200 g or 600 g in different trials and fell in about 0.5 s. Subjects prestiffened their arm muscles to absorb the impact at about 130 ms, irrespective of the specific mass of the ball. Instead, the amplitude of muscle activation scaled in proportion to the ball mass, because a stronger force is required to counteract a stronger ball momentum [29]. It has also been shown that the time of muscle contraction always leads the impact time by the same amount irrespective of the height of fall (Figure 6) [29]. Therefore, the motor system is accurately tuned to the effects of Earth gravity.

This tuning persists at the beginning of orbital flight, despite the sensory and cognitive evidence of weightlessness, and despite the motor responses being inappropriate to the new conditions [30]. These results are compatible with a Bayesian interpretation of the estimate of gravitational acceleration, if one assumes that the variance in the prior of 1 g acceleration is very small compared with the variance in the sensory likelihood. If so, the 1 g prior would bias strongly the estimate, until it is updated with prolonged exposure to weightlessness [31].

Gravity effects are taken into account not only by the motor system but also perceptually, as when people judge the duration of motion of a falling target [32, 33] or the period of oscillation of a pendulum [34]. Thus, in experiments in which a pendulum oscillates faster or slower than normal, the observers rate the oscillations violating the physical length-period relation as less natural than the oscillations complying with physics [34]. The implicit bias toward gravitational motion when viewing an oscillating pendulum is also revealed by the observation that harmonic motion is perceived as uniform [35]. Also, the perceptual judgment of passive egomotion along the vertical direction—simulated by means of immersive visual stimuli—is based on the internal model of gravity [36].

Just as in the case of the estimates of the direction of gravity, also those of visual gravitational acceleration generally depend on a combination of multiple cues, and such combination may obey Bayes' rules. The internal model provides the prior, while various sensory cues provide the likelihood of the estimate with a reliability that depends on the context. In one study, observers were asked to judge the duration of motion of a target accelerating in one of four different directions, downwards, upwards, leftwards, and rightwards, relative to a visual scene [33]. Downward motion complied with the gravity constraint, whereas motion in the other directions violated this constraint. Observers watched either a pictorial or an empty scene, while being upright or tilted by 45° relative to the monitor and Earth's gravity. In another condition, observers were upright and the scene was tilted by 45°. Discrimination precision was significantly better for downward motion than for the other directions. However, the difference in precision was not constant across conditions but was highest when both the observer and the pictorial scene were upright and lowest when the target direction in the empty scene was tilted by 45° relative to an upright observer. Thus, the behaviour observed in the study was consistent with the combination of pictorial cues, orientation of the observer relative to the physical vertical, and orientation of target motion relative to the physical vertical.

The interaction of the visual signals with vestibular signals about subject orientation relative to physical gravity was shown in a study performed during a parabolic flight campaign [37]. During each parabola, a 20 s weightless (0 g) phase was preceded and followed by 20 s of hypergravity (1.5–1.8 g). Strikingly, the timing of interception of a visual target moving along the visual vertical reversed sign during the weightless phases compared with the responses at normal gravity [37]. This reversal depends on the reversal of the otolith responses during the transition from hypergravity to hypogravity, which was sensed as a negative gravity, that is, as a gravitational pull in the upward direction (comparable to when we are suspended upside-down).

## 5. Neural Substrates of the Internal Model of Gravity Effects on Visual Motion

The hypothesis that the effects of gravity on a target motion are taken into account by combining multisensory information, including visual and vestibular cues, is supported by human neuroimaging studies. In a series of fMRI studies [7, 38–40], visual gravitational acceleration (involving either object motion or simulated egomotion) engaged a network of brain regions located within and around the Sylvian fissure close to the temporoparietal junction (TPJ): posterior insular cortex, retroinsula, parietal operculum, supramarginal gyrus, temporal operculum, and superior and middle temporal gyri. In addition, gravitational motion engaged primary somatosensory and motor cortex, ventral premotor cortex, SMA, cingulate cortex, visual cortex including the lingual gyrus, and several subcortical structures: posterior thalamus, putamen, cerebellum, and vestibular nuclei. A causal link between TPJ activity and the processing of visual gravitational motion has been demonstrated by transiently disrupting the activity of TPJ by means of transcranial magnetic stimulation (TMS) [41].

**Figure 7:**
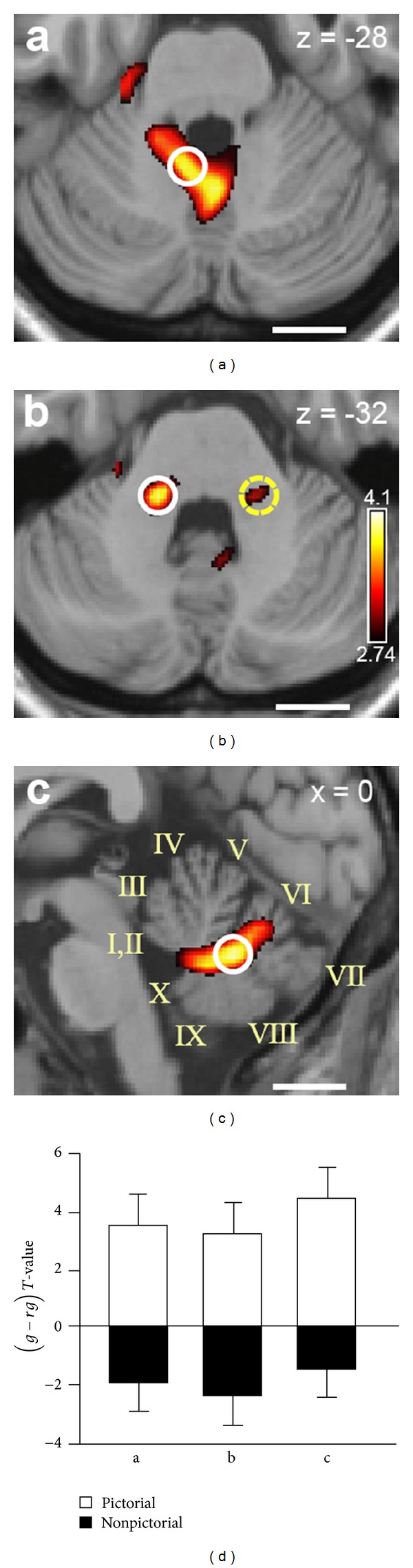
Functional magnetic resonance imaging of the cerebellum and brainstem in a task of interception of a ball moving along the vertical. Brain areas showing preferential activation for natural gravity motion specifically for the pictorial visual context. (a), (b), (c) Activations in the midline cerebellum ((a) axial section; (c) medial sagittal section) and vestibular nuclei ((b) axial section). Roman numerals in (c) denote Larsell lobules. White circles are centered on maximal statistical activation peaks. (a) Lobules IX/X. (b) Left vestibular nuclei. (c) Lobules VII/VIII. (d) Bar-graphs of the difference (± between-subjects s.e.m.)* t*-values for natural gravity (*g*) and artificial reversed gravity (*rg*) trials in pictorial (white) and nonpictorial (black) context for the activity peaks circled in (a), (b), and (c) (reproduced with permission from [7]).

As reviewed earlier, electrophysiological studies in the monkey showed that a population of Purkinje cells in the caudal cerebellar vermis encodes head tilt, thus reflecting an estimate of gravity direction based on vestibular information [5]. Interestingly, human posterior cerebellar vermis (a homologue region of that studied by Laurens et al. [5] in monkeys) and vestibular nuclei appear to be involved in combining pictorial information with the internal model of gravity to extract gravitational motion from visual scenes (Figure 7) [7].

In sum, the neuroimaging studies reviewed above indicate that the effects of gravity on visual motion are encoded in a highly distributed cortical-subcortical network. Several regions of this network colocalize with the regions independently activated by vestibular caloric stimuli [38]. These regions then presumably belong to the multimodal vestibular network, which also responds to visual and neck proprioceptive stimuli [42, 43]. Lesions of vestibular cortex can lead to a tilt of the perceived visual vertical and rotational vertigo/unsteadiness [44], while focal electrical stimulation or epileptic discharges can elicit sensations of self-motion or altered gravity [45, 46].

## 6. Conclusions

We argued that an apparently simple problem such as that of monitoring gravity effects on our body and on the external environment is in fact computationally very demanding, even for a high-powered brain such as that of primates. Measurements derived from individual sensory organs are often ambiguous (due to the intrinsic constraints of physical laws) and noisy (due to biological limitations). However, the combination of multisensory signals (visual, vestibular, proprioceptive, and visceral) and the reliance on internal models of physics yield estimates which are very accurate under normal conditions, but which can fail badly under anomalous conditions (such as the early phases of space flight). Central processing of multisensory information and internal models occurs in a widely distributed network of cortical and subcortical regions. The extensive integration of sensory and motor information in this network makes gravity-related information available to many vital functions of the organism.
